# Long-acting injectables for global health: drug development considerations and applications in malaria and tuberculosis

**DOI:** 10.12688/gatesopenres.16401.1

**Published:** 2026-10-14

**Authors:** Long-acting Injectable Workshop Group, Fran Berlioz-Seux, Beno�t Bestgen, Jeremy Burrows, Tomas Cihlar, Zedong Dong, Debra Flood, Nathalie Gobeau, Ren� Holm, Paul Jackson, Linu John, Mike Morimoto, John Pottage, Hanu Ramachandruni, Rada Savic, Bill Spreen, Rajneesh Taneja, Belen Tornesi, Birgit Verstynen, Peter Warner, Charles Wells, Paul Willis, Ping Zhao, Fran Berlioz-Seux, Benoît Bestgen, Jeremy Burrows, Tomas Cihlar, Zedong Dong, Debra Flood, Nathalie Gobeau, René Holm, Paul Jackson, Linu John, Mike Morimoto, John Pottage, Hanu Ramachandruni, Rada Savic, Bill Spreen, Rajneesh Taneja, Belen Tornesi, Birgit Verstynen, Peter Warner, Charles Wells, Paul Willis, Ping Zhao

**Affiliations:** 1 Bill & Melinda Gates Foundation, Seattle, Washington, USA; 2 MMV Medicines for Malaria Venture, Geneva, Switzerland; 3 Liverpool School of Tropical Medicine, Liverpool, England, UK; 4 Gilead, San Mateo, CA, USA; 5 Bill & Melinda Gates Medical Research Institute, Cambridge, Massachusetts, USA; 6 University of Southern Denmark, Odense, Denmark; 7 University of California San Francisco, San Francisco, California, USA; 8 Intrepid Alliance, Philadelphia, PA, USA; 9 TB Alliance, New York, NY, USA; 10 Independent Toxicology Consultant, Antwerp, Belgium

**Keywords:** long-acting injectables, malaria, tuberculosis, drug discovery, drug development, global health, neglected diseases.

## Abstract

Long-acting injectable (LAI) drug formulations represent a transformative modality in modern therapeutics, delivering medications that maintain therapeutic effects over extended periods. While initially developed to improve patient adherence and acceptability for applications such as hormonal contraception and chronic conditions, including psychiatric disorders, their recent success in HIV treatment and prevention has catalyzed a growing interest in their application to global health priorities. While there are effective oral drug regimens available for tuberculosis (TB) treatment and malaria chemoprevention, their dosing regimens challenge patient adherence. By providing sustained pharmacological activity over extended periods, LAIs decouple therapeutic efficacy from patient adherence and simplify drug dosing regimens, enhancing real-world effectiveness. This position paper synthesizes the emerging consensus on the application of LAIs for global health from a multidisciplinary expert workshop convened by Medicines for Malaria Venture (MMV) and the Gates Foundation in Chicago on March 17–19, 2026. Contributions considered compound selection, formulation design, pharmacokinetic and pharmacodynamic requirements, safety and tolerability profiles, toxicology packages for first-in-human studies, regulatory pathways, and the challenges of human dose prediction. Emphasis was placed on the importance of preclinical-to-clinical translation, particularly through the application of pharmacometric tools and model-informed drug development. The workshop highlighted strategic priorities for advancing the LAI field, including coordinated cross-sector approaches, pre-competitive data sharing, and communities of practice to address critical knowledge gaps. These actions aim to realize the potential for expanding LAI technologies to advance global health.

**Abbreviations**
HIV: human immunodeficiency virus
LAI: long-acting injectable
MIDD: model-informed drug development
MMV: MMV Medicines for Malaria Venture
PrEP: Pre-exposure prophylaxis
TB: tuberculosis
TBDA: Tuberculosis Drug Accelerator
TPP: target product profile

## 1. Introduction

Tuberculosis (TB) and malaria are global health priorities. ^1^ In 2024, there were 282 million malaria cases and 10.7 million people became ill with TB, while an estimated 2 billion people are infected with *Mycobacterium tuberculosis.* ^2^ ^,^ ^3^ Malaria causes nearly 600,000 deaths annually, while TB causes approximately 1.25 million deaths each year worldwide. ^2^ ^,^ ^3^ The World Health Organization has set ambitious targets to decrease TB deaths by 90% and incidence by 80%, and malaria deaths and incidence by 90% by 2030 compared with 2015. ^4^ Despite significant progress towards these goals, major advancement is hampered by the limitations of the available tools.

Long-acting injectable (LAI) drugs and their formulations present a compelling alternative to conventional daily oral therapies, simplifying treatment regimens and improving adherence. The success of antiretroviral LAIs confirmed the potential benefits of LAIs in infectious diseases. Replacing daily pills with cabotegravir/rilpivirine injections every 2 months was a landmark advance for HIV treatment, improving adherence and reducing stigma. ^5^ ^–^ ^7^ Subsequently, the approval of twice-a-year injections with lenacapavir for pre-exposure prophylaxis (PrEP) was transformative, especially for those struggling with adherence, lack of privacy, or with limited access to healthcare services. ^8^ Lenacapavir is now being evaluated as a once-yearly PrEP option, supported by advanced modeling techniques, including preclinical studies, imaging, and computational simulations. ^9^ Given these advances, the global health community is now asking whether the same benefits achieved for people living with HIV and those in need of HIV PrEP are achievable for malaria chemoprevention and TB treatment and prevention.

Translating the success of LAIs achieved with HIV treatment and PrEP to the prevention and treatment of TB and the prevention of malaria introduces challenges. These include the complexities of pathogen biology, specific stringent drug profile requirements for potency, stability and formulation, as well as diverse healthcare delivery contexts and ensuring global access. Moreover, in addition to repurposing drugs already characterized in human oral drug trials, programs are increasingly advancing new chemical entities with both oral and LAI formulations and, in the case of malaria, directly into LAI preparations.

This position paper outlines the key discussions and recommendations from a multidisciplinary expert workshop convened by Medicines for Malaria Venture (MMV) and the Gates Foundation in Chicago on March 17–19, 2026. The meeting brought together more than forty specialists to consolidate learnings, identify knowledge gaps, and generate recommendations to accelerate and scale the development of transformational LAI medicines in TB and malaria.

## 2. LAIs as a transformative modality for TB and malaria

Malaria and TB disproportionately affect low- and middle-income countries. Prevention and treatment with oral medicines demand adherence levels that are often difficult to sustain. ^2^ ^,^ ^3^ Sub-optimal adherence undermines preventive efficacy, contributes to treatment failure, drives ongoing transmission, and increases the risk of drug resistance selection. ^2^ ^,^ ^3^ In TB, standard treatment regimens commonly require at least six months of daily therapy, with directly observed therapy for five days each week throughout the treatment course. ^10^ These extended, supervised dosing regimens impose a substantial burden on health care systems, with adverse economic and social impacts for patients. ^11^ Malaria chemoprevention requires repeated monthly dosing, aiming to protect high-risk populations such as children and during pregnancy. ^12^ Given the high prevalence of malaria and the extent of the populations at risk, effective deployment of chemoprevention is a major public health activity in endemic regions. ^13^

By providing sustained pharmacological activity over extended periods, LAIs reduce dependence on daily adherence and simplify drug regimens, thereby enhancing real-world effectiveness. Patient and provider preference studies evaluating putative TB and malaria LAI products indicate strong acceptance of LAI approaches, with convenience and reduced treatment burden emerging as key advantages. ^14^ ^,^ ^15^

Target product profiles (TPPs) define the desired characteristics of LAI products, including the duration of protection, dosing frequency, safety requirements, and cost constraints ( Figure 1). The TB Alliance, the TB Drug Accelerator (TBDA), the Gates Foundation, and the PAN-TB Partnership are closely linked organizations working to accelerate the development of new TB treatments. Together, they have aligned around a shared TPP with characteristics including shorter treatment duration, effectiveness against both drug-sensitive and drug-resistant TB, improved safety, and global accessibility. The WHO has also released a draft target regimen profile for public consultation. ^16^ In this context, the LAI strategy envisions a 1–2-month oral induction phase followed by LAIs providing at least 2 months of effective treatment for a maximum treatment duration (oral plus LAIs) of 4 months ( Figure 1). This would substantially reduce the adherence burden compared to standard treatments. ^10^ However, the optimal position is to achieve a single-instance therapy using a combination of LAIs and to extend the use case from treatment to addressing asymptomatic and latent infection as well as prevention.

**Figure 1.**
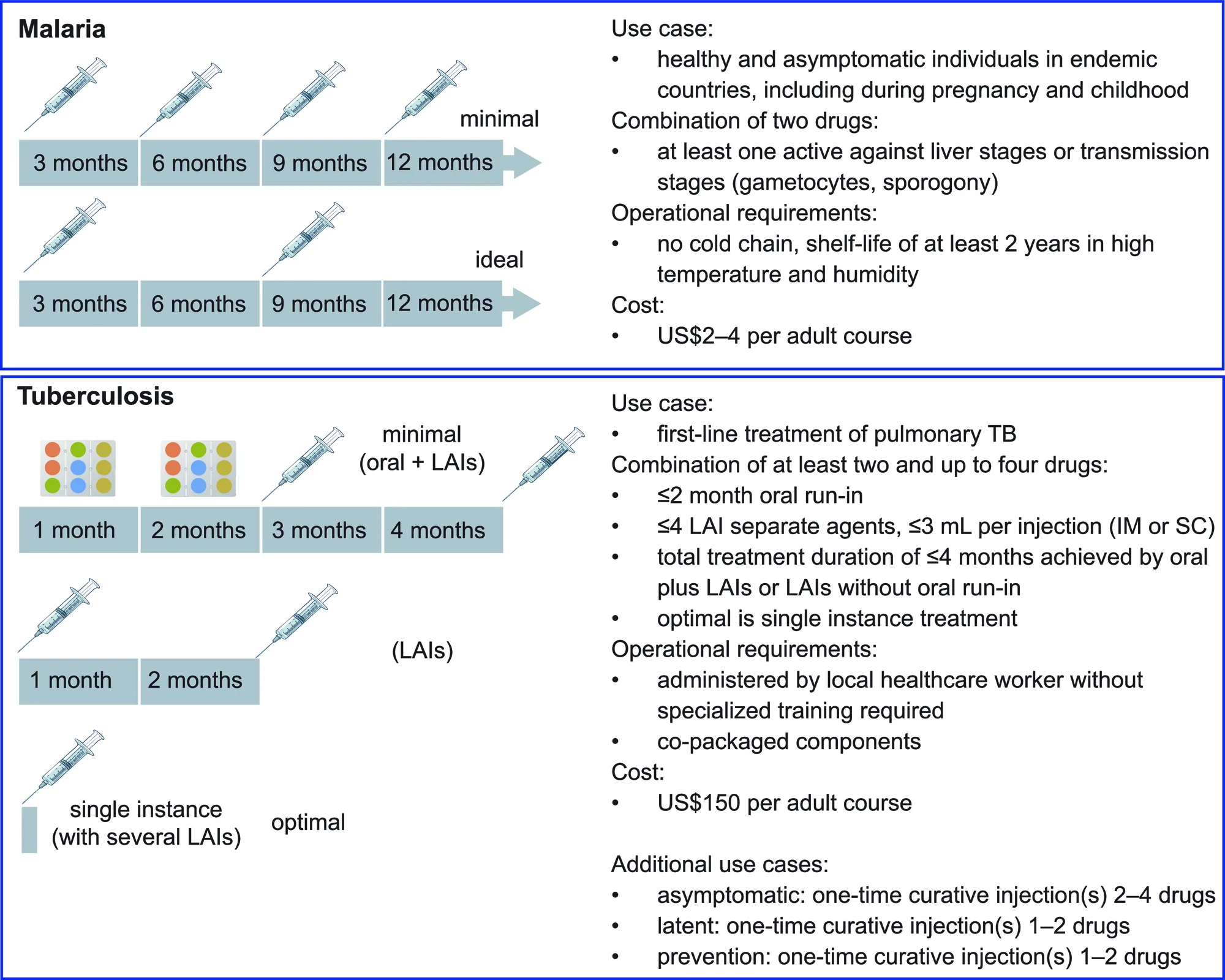
Target product profiles illustrate the potential for LAIs to dramatically reduce the barriers to adherence for malaria and TB, improving patient health outcomes and reducing transmission. LAI, long-acting injectable; IM, intramuscular; SC, subcutaneous; TB, tuberculosis.

In malaria, MMV’s TPP seeks to extend chemoprevention to address the asymptomatic transmission reservoir. ^13^ In this case, the chemopreventive product would be widely deployed in communities to treat asymptomatic infections and protect against further infection. This strategy aims to both protect individuals and reduce transmission at the population level, decreasing disease burden and supporting malaria elimination. Protection duration would be between three and six months ( Figure 1), with tolerability enabling broad implementation and safety profiles consistent with use in pregnancy and childhood. ^13^

For both TB and malaria, combination therapy is anticipated to reduce the risk of developing resistance selection from monotherapy exposure. In TB, the current hypothesis is that at least two drugs with long half-lives must be present in caseous lesions simultaneously throughout the dosing interval to kill the tolerant sub-populations that are likely to be responsible for prolonging disease and leading to relapse. ^16^ Accordingly, there is a need for lesion-penetrating, highly potent new chemical entities for the continuation-phase LAI backbone, while the oral induction phase can incorporate bactericidal agents with shorter half-lives that may not be suitable for LAI. In the case of malaria, MMV’s TPP ideally requires a two-drug LAI combination, ideally co-formulated rather than separate injections. ^13^ To protect against resistance selection, the pharmacological duration of cover for the components should be matched. However, matching the pharmacological duration of two LAI drugs within a combination is technically demanding and may be unachievable with the available molecules. In such a scenario, it is required that the partner drug with a longer pharmacokinetic profile has a high barrier to resistance.

## 3. Compound properties and LAI platforms

Successful LAI development depends on identifying compounds with a combination of high potency, low systemic clearance, and physicochemical properties supporting sustained release at optimal rates to cover the desired dosing intervals. Low aqueous solubility, often considered undesirable in oral drug development, is advantageous in LAI systems as it prolongs depot retention. However, these relationships are not absolute, and compound behavior is strongly influenced by formulation design and biological context. Other factors to consider include low irritation potential, high drug loading, particle size constraints, and injectability with a 22G to 25G needle, maintenance of sterility, avoidance of the need for a cold chain, and the need to maintain stability in tropical climates. In some cases, the target physicochemical properties to achieve an optimal release rate from the injection site depot while minimizing injection site reactions could be achieved by various prodrug strategies.

LAIs exhibit ‘flip-flop’ pharmacokinetics, where the absorption kinetics from the injection site, rather than systemic elimination, determine the compound persistence in the body. This introduces significant translational uncertainty in preclinical dose prediction, as the drug release rate and pharmacokinetic profile can be influenced by formulation design, particle size, form (crystalline or amorphous), tissue perfusion, and local immunological responses. ^17^ ^,^ ^18^

Formulation science is central to LAI success. Diverse platforms exist, including aqueous suspensions and polymer-based depots, such as *in situ* forming gelling systems. ^19^ ^–^ ^22^ Each formulation technology offers trade-offs in scalability, drug loading, cost of goods, and release control. The route of administration further influences performance. Intramuscular tissue is more vascularized and can usually accommodate larger injection volumes per administration. Subcutaneous tissue is less perfused and more sensitive to irritants as well as sometimes undesirable morphological changes such as temporary nodule formation. Overall, no single platform is universally optimal. Selection is guided by the physicochemical properties of the individual active pharmaceutical ingredients, dose requirements, administration route, desired release profile, manufacturability (including cost and scale-up potential), and the clinical context, as well as patient and provider preference. Importantly, key formulation decisions should ideally be made early in the development process, as late-stage changes can significantly alter pharmacokinetics and safety, requiring additional studies to be performed, generating delays and additional development costs.

## 4. Translational and pharmacometric approaches

The most widely discussed knowledge gap at the workshop was the difficulty of translating preclinical LAI pharmacokinetics to humans. Predicting human LAI-specific properties (bioavailability and absorption rate) remains an unresolved challenge in LAI development, requiring integrated approaches and management of uncertainty. ^20^ In oral programs, translation of absorption from animal/in vitro data is widely established, supported by extensive data availability. ^23^ In contrast, LAIs lack a robust historical translation framework and preclinical species do not necessarily predict human LAI release profiles. Workshop participants recommended presenting dose predictions as a range of scenarios spanning best- and worst-case assumptions of absorption kinetics. Therefore, multiple formulations, with varying particle size, excipient composition, or drug loading, should be examined in preclinical studies to ensure that if the primary formulation underperforms, a contingency option can be activated without incurring a substantial delay to the program. Alternatively, to de-risk and accelerate a program, at least two LAI formulations may need to be advanced into the clinic in parallel. However, this can markedly increase development costs and create operational challenges, particularly in a global health setting.

Model-informed drug development (MIDD) is a key framework that supports both drug development and regulatory decision-making. ^24^ It enables quantitative, data-driven predictions that improve hypothesis testing and streamline the evaluation of drug candidates, thereby reducing risk for the costly late-stage studies. ^25^ MIDD was highlighted as an essential tool for predicting the behavior of LAIs in humans. ^20^ ^,^ ^26^ ^,^ ^27^ LAIs exhibit release kinetics that depend on complex factors, such as the molecule properties, formulation, particle size, dose and volume administered, and tissue environment. Therefore, translating preclinical findings to humans requires sophisticated models that integrate drug properties, formulation characteristics, and physiological variables. Key MIDD applications for LAI development include estimating dose requirements, optimizing dosing intervals, and evaluating the risk of subtherapeutic “tail” exposures that may promote resistance emergence. Both MMV and the TBDA have built pharmacometric tools to support compound and product development, primarily for orally administered therapies. These tools are now being adapted and extended to support the development of LAI formulations.

### 4.1 Malaria MIDD tools

In malaria, two tools have been developed specifically to help discovery teams accelerate the delivery of high-quality candidates for oral chemoprevention – MMVFree and MMVSola. For chemoprevention, early dose prediction focuses on maintaining a human predicted systemic unbound plasma drug concentration above the unbound parasiticidal minimum inhibitory concentration (the lowest drug concentration that prevents net growth of asexual blood-stage malaria parasites) over the required duration of protection. MMVFree is used early in the discovery process to guide optimization of a series toward a lower clinical chemopreventive dose. MMVSola is intended for later stages of discovery once a lead compound has been identified and is being evaluated for potential clinical progression. These tools are now being extended to support the development of LAI formulations by incorporating absorption-rate considerations, enabling the assessment of how formulation-dependent absorption kinetics influence drug exposure, duration of protection, and the feasibility of achieving TPP requirements.

### 4.2 Tuberculosis MIDD tools

The application of MIDD to oral TB treatment has focused on the preclinical to clinical stages of development and has the potential to significantly compress drug development timescales without compromising scientific rigor. ^24^ In parallel, TB drug development benefits from a highly integrated and collaborative data ecosystem, in which preclinical, translational, and clinical datasets are shared across public–private partnerships and global consortia, enabling end-to-end regimen optimization from molecular design through Phase III evaluation. This has enabled the development of robust, validated modeling tools to support decision-making, dose optimization, and predictive analytics. These tools are continuously improved as more data are incorporated. An integrated platform has been built to support the entire development process, comprising clinical trial risk algorithms to predict Phase III outcomes, ^28^ ^,^ ^29^ a lesion penetration model to characterize drug efficacy in target tissues, ^30^ systems pharmacology models to add mechanistic understanding of immune response and disease biology, ^31^ relapse prediction tools using machine learning on early biomarker data, ^32^ and a translational pharmacokinetic-pharmacodynamic platform to predict monotherapy and combination therapy Phase II dose–response. ^33^ At its intersection is a human dose prediction tool, which incorporates plasma pharmacokinetics, site-of-disease efficacy, and early potency data to predict an efficacious dose range that can enable Phase III success. Overall, this prediction tool can estimate dose, early bactericidal efficacy, and clinical endpoints such as time to sputum conversion. This tool could also be extended to help the preclinical development of LAI products, with a likely significant reduction in timescales for clinical development. However, predictive performance depends heavily on data quality and the availability of well-characterized reference compounds, requiring open collaboration and extensive data generation.

## 5. Safety, toxicology, and regulatory considerations

LAI development introduces distinctive safety and tolerability considerations due to the prolonged systemic exposure. Thus, the risk:benefit balance must be closely examined. This is especially important when considering administration to young children and women, especially in areas of the world where pregnancy status may be unknown or not disclosed. Robust preclinical and clinical safety data are needed while generating the necessary information to ensure equitable access. ^34^ ^–^ ^36^

One strategy emphasizes oral-first approaches to obtain initial clinical data on the molecule, minimizing the potential for unforeseen safety concerns in LAI programs. ^37^ ^,^ ^38^ However, in some cases, direct LAI development is supported by prior clinical or preclinical evidence, such as known tolerability profiles, established pharmacokinetics, or well-characterized mechanisms of action within a compound class. First-in-human studies may use oral administration to characterize pharmacokinetics and safety parameters. However, the direct clinical testing of LAIs without prior data from oral administration may be considered if supported by a robust non-clinical safety and pharmacokinetic package or if the new molecule does not support oral dosing. ^37^ ^,^ ^39^ Injection site reactions are a key safety endpoint for LAIs, requiring dedicated evaluation in appropriate animal models followed by early clinical studies. Regulatory pathways increasingly emphasize early characterization of advanced formulations before first-in-human studies.

## 6. Strategic priorities and recommendations

A central conclusion of the workshop was that LAI development should be conducted as an integrated, system-level parallel optimization goal rather than a sequential process ( Figure 2). The primary translational challenge is the poor predictability of human LAI absorption rate and bioavailability from preclinical models. Thus, key priorities need to include early integration of formulation and pharmacokinetic considerations into drug design decision-making, development of predictive frameworks for absorption rate and bioavailability, tolerability assessment, and early engagement with regulatory authorities on LAI strategies. Equally important is the establishment of pre-competitive data sharing mechanisms to enable collective learning from both successful and failed compounds. Close contacts among communities of practice spanning TB, malaria, and HIV are essential for accelerating cross-disease translation and reducing duplication of effort. Finally, sustained investment in translational modeling infrastructure is required to support iterative decision-making and reduce clinical attrition. This includes expansion of human pharmacokinetic datasets (both successful and failed LAI formulations), improved disease models (e.g., lesion and tissue distribution models for TB, and liver-stage models for malaria), and better integration of systems pharmacology with clinical trial design.

**Figure 2.**
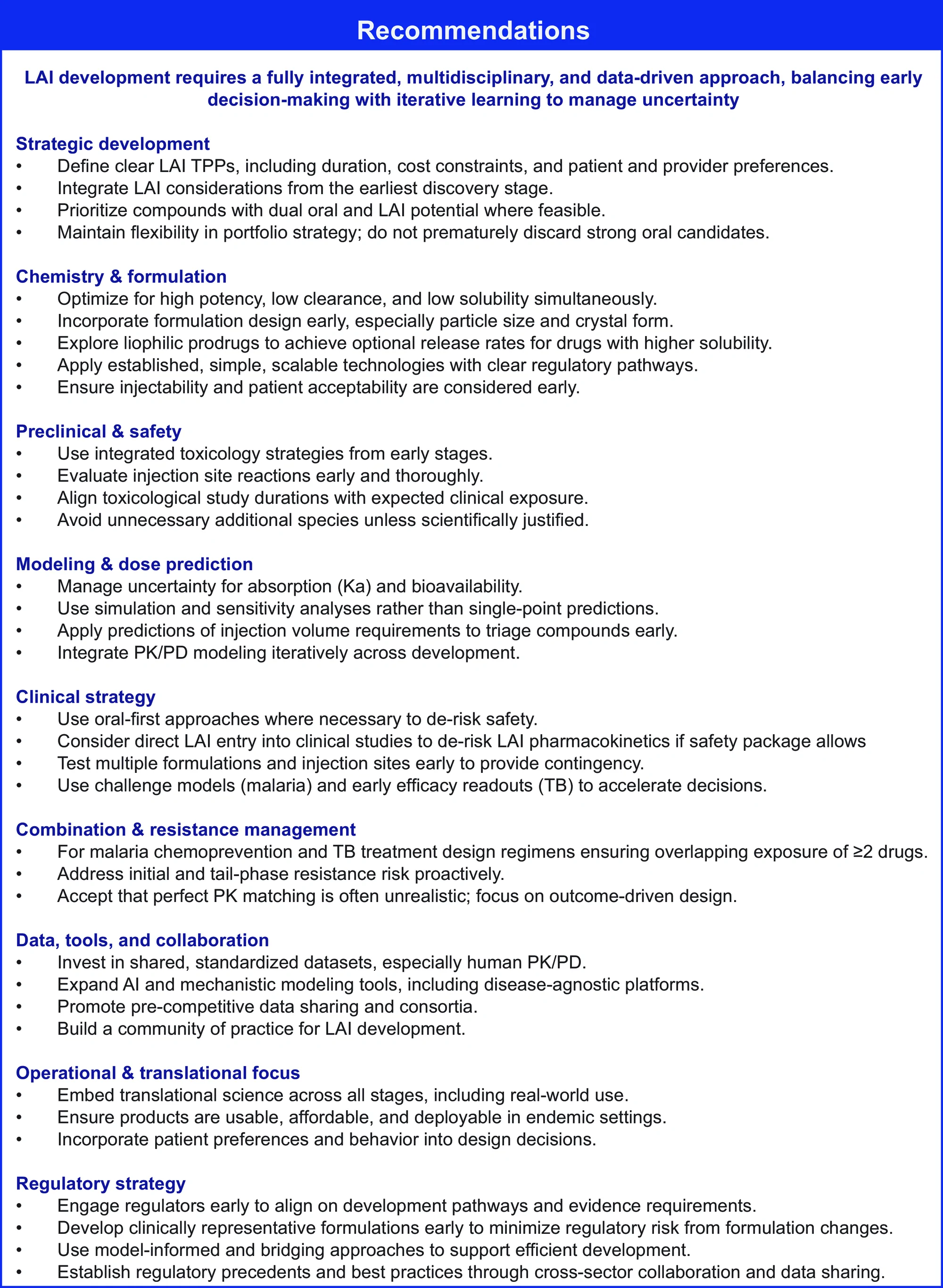
Summary of recommendations. AI, artificial intelligence; Ka, absorption rate constant; LAI, long-acting injectable; PD, pharmacodynamic; PK, pharmacokinetics; TB, tuberculosis; TPP, target product profile.

## 7. Conclusion

LAIs offer a credible and transformative approach for improving the treatment and prevention of TB and the prevention of malaria. By shifting the focus from daily oral dosing to sustained exposure, LAIs can simplify regimens, improve adherence, and improve intervention outcomes, particularly in settings where healthcare access is limited. However, at the same time, LAIs present technical challenges that introduce new complexities into drug and formulation discovery, development, and implementation which require coordinated, multidisciplinary collaboration across scientific and technical functions. Moreover, patient acceptability and access must be considered early in development.

Foundational science and experience from other therapeutic areas have matured sufficiently to support the development and ultimately the regulatory licensure of LAIs for TB and malaria, but success will depend on coordinated, cross-sector efforts to address persistent gaps in data, prediction, and translational alignment. With appropriate investment in shared frameworks and collaborative structures, LAIs have the potential to significantly accelerate global progress to end TB and eliminate malaria.

## Author contributions

All authors were involved in conceptualization, writing – original draft and writing – review and editing. All authors reviewed the final version of the manuscript and approved the submission.

## Disclaimer

The views expressed in this article are those of the author(s). Publication in Gates Open Research does not imply endorsement by the Gates Foundation.

## Data Availability

No data are associated with this article.
